# Distribution of *espM* and *espT* among enteropathogenic and enterohaemorrhagic *Escherichia coli*

**DOI:** 10.1099/jmm.0.010231-0

**Published:** 2009-08

**Authors:** Ana Arbeloa, Miguel Blanco, Fabiana C. Moreira, Richard Bulgin, Cecilia López, Ghizlane Dahbi, Jesús E. Blanco, Azucena Mora, María Pilar Alonso, Rosalia Ceferina Mamani, Tânia A. T. Gomes, Jorge Blanco, Gad Frankel

**Affiliations:** 1 Centre for Molecular Microbiology and Infection, Division of Cell and Molecular Biology, Imperial College London, London SW7 2AZ, UK; 2 Laboratorio de Referencia de *E. coli*, Departamento de Microbiología y Parasitología, Facultad de Veterinaria, Universidad de Santiago de Compostela, Lugo, Spain; 3 Departamento de Microbiologia, Imunologia e Parasitologia, Universidade Federal de São Paulo, São Paulo, Brazil; 4 Unidade de Microbioloxía Clínica, Complexo Hospitalario Xeral-Calde, Lugo, Spain

## Abstract

Enterohaemorrhagic *Escherichia coli* (EHEC) and enteropathogenic *E. coli* (EPEC) translocate dozens of type III secretion system effectors, including the WxxxE effectors Map, EspM and EspT that activate Rho GTPases. While *map*, which is carried on the LEE pathogenicity island, is absolutely conserved among EPEC and EHEC strains, the prevalence of *espM* and *espT* is not known. Here we report the results of a large screen aimed at determining the prevalence of *espM* and *espT* among clinical EPEC and EHEC isolates. The results suggest that *espM*, detected in 51 % of the tested strains, is more commonly found in EPEC and EHEC serogroups that are linked to severe human infections. In contrast, *espT* was absent from all the EHEC isolates and was found in only 1.8 % of the tested EPEC strains. Further characterization of the virulence gene repertoire of the *espT*-positive strains led to the identification of a new *ζ*2 intimin variant. All the *espT*-positive strains but two contained the *tccP* gene. *espT* was first found in *Citrobacter rodentium* and later *in silico* in EPEC E110019, which is of particular interest as this strain was responsible for a particularly severe diarrhoeal outbreak in Finland in 1987 that affected 650 individuals in a school complex and an additional 137 associated household members. Comparing the protein sequences of EspT to that of E110019 showed a high level of conservation, with only three strains encoding EspT that differed in 6 amino acids. At present, it is not clear why *espT* is so rare, and what impact EspM and EspT have on EPEC and EHEC infection.

## INTRODUCTION

Enterohaemorrhagic *Escherichia coli* (EHEC) comprise a subgroup of Shiga-toxin producing *E. coli* that can cause bloody diarrhoea, haemorrhagic colitis and haemolytic-uraemic syndrome (reviewed by Tarr, 1995). EHEC O157 : H7 is the most common and virulent serotype that is implicated worldwide in human disease, although non-O157 EHEC serotypes, particularly O26, O103, O111, O118 and O145, are also prevalent (reviewed by Nataro & Kaper, 1998). Enteropathogenic *E. coli* (EPEC) is the leading cause of mortality due to infantile diarrhoea in the developing world (reviewed by Chen & Frankel, 2005). EPEC strains comprise a diverse group of serotypes that may be divided into typical EPEC (tEPEC) and atypical EPEC (aEPEC) based on the presence or absence of a large virulence plasmid (EAF), respectively (Kaper, 1996)

EPEC and EHEC, as well as the mouse pathogen *Citrobacter rodentium* (reviewed by Mundy *et al.*, 2005), colonize the gut mucosa via attaching and effacing (A/E) lesions, which are characterized by the close association of the bacteria with the enterocyte plasma membrane and localized breakdown of the brush border microvilli (Knutton *et al.*, 1987; reviewed by Frankel & Phillips, 2008). The ability to induce A/E lesions is associated with the LEE pathogenicity island, which encodes gene regulators, intimin (Jerse *et al.*, 1990), a type III secretion system (Jarvis *et al.*, 1995), chaperones, translocator and effector proteins (reviewed by Garmendia *et al.*, 2005a). The principal effector protein needed for A/E lesion formation is Tir, which, once translocated, is integrated into the mammalian cell plasma membrane where it serves as a receptor for intimin (Kenny *et al.*, 1997). Recent studies have shown that different EPEC and EHEC strains encode distinct intimin and Tir types; currently there have been 27 intimin and 8 Tir types reported (Blanco *et al.*, 2006a, b; Garrido *et al.*, 2006; J. Blanco, unpublished data).

EPEC E2348/69, which is the prototype strain used worldwide to study EPEC infection, encodes 21 LEE and non-LEE effectors (Iguchi *et al.*, 2009). Other EPEC strains encode a greater number of T3SS effectors: 28 in EPEC B171 (Ogura *et al.*, 2008), 40 in EPEC E22 and 24 in EPEC E110019 (Iguchi *et al.*, 2009). EHEC O157 Sakai encodes 50 effectors, representing the most complex repertoire among A/E pathogens (Tobe *et al.*, 2006). This shows that the A/E pathogen class is much more heterogeneous than was previously thought, comprising strains expressing unique complements of T3SS effector proteins and as a result employing diverse infection strategies.

A novel family of T3SS effectors, known as the WxxxE proteins, was recently described (Alto *et al.*, 2006), which include IpgB1 and IpgB2 from *Shigella*, SifA and SifB from *Salmonella*, and Map (Kenny & Jepson, 2000), EspM (Arbeloa *et al.*, 2008) and EspT (Bulgin *et al.*, 2008) from EPEC and EHEC. Members of the WxxxE family are important virulence factors. For example, SifA is essential for intracellular *Salmonella* survival (Beuzon *et al.*, 2000) and IpgB1 is essential for *Shigella* cell invasion (Ohya *et al.*, 2005). Map, which is encoded on the LEE pathogenicity island and is absolutely conserved among EPEC and EHEC strains, plays a role in colonization *in vivo* (Mundy *et al.*, 2004) and triggers transient filopodia by activating the Rho GTPase Cdc42 (Kenny *et al.*, 2002; Berger *et al.*, 2009). The different EspM variants induce formation of stress fibres by activating RhoA (Arbeloa *et al.*, 2008), while EspT from the *C. rodentium* induces formation of extensive lamellipodia by activation of Rac-1 and Cdc42 (Bulgin *et al.*, 2009). By sequence homology searches we recently identified homologues of *espM* and *espT* in EPEC strain E110019 (Bulgin *et al.*, 2009), a clinical isolate from a diarrhoeal outbreak in Finland in 1987 (Viljanen *et al.*, 1990). EspM_E110019_ is 100 % identical to the EspM of EHEC O157 Sakai, while EspT_E110019_ shares 79 % sequence homology with the *C. rodentium* EspT, including the WxxxE motif. The aim of this study was to determine the prevalence of *espM* and *espT* among clinical EPEC and EHEC isolates.

## METHODS

### Bacterial strains.

The bacterial strains used in this study included EPEC strains E2348/69 (Levine *et al.*, 1978) and E110019 (Viljanen *et al.*, 1990), EHEC O157 : H7 strain Sakai (Hayashi *et al.*, 2001), *C. rodentium* strain ICC169 (Barthold *et al.*, 1976; Wiles *et al.*, 2004), and 932 clinical EHEC and EPEC isolates.

### Serotyping.

The determination of O and H antigens was carried out using the method described by Guinée *et al.* (1981) employing all available O (O1–O185) and H (H1–H56) antisera. All antisera were obtained and absorbed with the corresponding cross-reacting antigens to remove the non-specific agglutinins. The O antisera were produced in the Laboratorio de Referencia de *E. coli* and the H antisera were obtained from the Statens Serum Institut, Copenhagen, Denmark.

### Prevalence of *espT* and *espM* among clinical EPEC and EHEC strains.

In order to screen for *espM* by PCR we used the eight *espM* sequences identified in EHEC O157 strain Sakai, EPEC strains B171 and E22, and *C. rodentium* to design common internal espM-1 (5′-TCTTTCAGCTCTTTTGGTAT-3′) and espM-2 (5′-CCAAAAGAAGCATTCCCCATTAT-3′) forward and reverse primers (30 cycles of 94 °C for 45 s, 48 °C for 1 min and 72 °C for 1 min). The identity of representative PCR amplicons was confirmed by DNA sequencing. A second round of PCR was employed to screen representative espM-1 and espM-2 PCR negative strains using primers espM-3 (5′-TGATGAGGTCATGAAATGTTCAAT-3′) and espM-4 (5′-ATGATTAATAGAACTTTG-3′) (30 cycles of 94 °C for 45 s, 50 °C for 1 min and 72 °C for 1 min).

We used the *espT* sequences from *C. rodentium* and E110019 to design internal espT-1 (5′-AATCTCATTCTCTTATC-3′) and espT-2 (5′-TCATGTGATGAGTGGATG-3′) primers for PCR (30 cycles of 94 °C for 45 s, 55 °C for 1 min and 72 °C for 1 min).

A further three rounds of PCR were employed to screen representative espT-1 and espT-2 PCR-negative strains using additional common internal primers espT-3 (5′-ATAGATGGTTTCTTTTTAGG-3′) and espT-4 (5′-CATCCAACGAGAAACCGCAAT-3′), and primers espT-5 (5′-CCGgaattcATGCCGGGAACAATAAGCTCCAG-3′) and espT-6 (5′-GGGAAGCTTTTAGGTTCTCTGAGCCTC-3′) and espT-7 (5′-TTGAATTCATGCATAGCATGCCAGGA-3′) and espT-8 (5′-CCAATGCACTGCAGGGAGCATTAAACATATTTTAAATTTCTC-3′), which correspond to the 5′ and 3′ ends of the *C. rodentium* and E110019 *espT* homologue, respectively (30 cycles of 94 °C for 45 s, 52 °C for 1 min and 72 °C for 1 min).

EHEC O157 : H7 Sakai, *C. rodentium* and EPEC E2348/69 were used as positive and negative controls.

### Intimin, Tir and TccP typing of the *espT*-positive strains.

Intimin and Tir typing was performed by PCR and sequencing as previously described (Blanco *et al.*, 2006b; Garrido *et al.*, 2006). The nucleotide sequence of the amplification products purified using a QIAquick DNA purification kit (Qiagen) was determined by the dideoxynucleotide triphosphate chain-termination method of Sanger, with a BigDye terminator v3.1 cycle sequencing kit and an ABI 3100 genetic analyzer (Applied Biosystems). The new *eae* sequences of the strains analysed were deposited in the European Bioinformatics Institute EMBL nucleotide sequence database. The presence of *tccP2* was determined by PCR as described by Whale *et al.* (2007).

## RESULTS AND DISCUSSION

### Screening O157 and non-O157 EHEC strains for the presence of *espM* and *espT*

We determined the prevalence of *espM* and *espT* among 45 non-sorbitol fermenting (non-SF) EHEC O157 : H7 (expressing VT1 and VT2), isolated in Spain, Canada and Bolivia, and two SF EHEC O157 : H- (expressing VT2), isolated in Germany. *espM* was found in 43 of the non-SF O157 isolates (96 %) and in the 2 SF isolates. We then screened 151 non-O157 EHEC strains. *espM* was found in 60 of the 62 (97 %) non-O157 EHEC strains belonging to serogroups O26, O103, O111, O118 and O145 (Table 1). In contrast *espM* was found in only 17 of 89 (19 %) strains belonging to the other non-O157 serogroups (Table 1). All the O157 and non-O157 strains were *espT* gene negative.

In order to confirm the absence of *espM* and *espT* from the PCR gene-negative strains, we screened 50 O157 and non-O157 EHEC strains with a second set of conserved *espM* primers (espM-3 and espM-4) and three sets of *espT* primers (espT-3 and espT-4, espT-5 and espT-6, and espT-7 and espT-8). All the isolates remained *espM* and *espT* gene negative in these tests.

### Screening tEPEC and aEPEC isolates for the presence of *espM* and *espT*

We screened a total of 132 tEPEC strains, isolated in Brazil, Bolivia, Burundi, Spain, Chile, Germany, the UK and Uruguay, and 602 aEPEC strains, isolated in Brazil, Bolivia, Chile and Spain, for the presence of *espM* and *espT*. *espM* was found in 91 tEPEC isolates (69 %) belonging to 16 different serogroups (the O serogroup of 6 strains was non-typable – ONT) (Table 2). *espM* was found in 258 aEPEC isolates (43 %) belonging to 59 different serogroups [the O serogroup of 109 strains was ONT and of 2 isolates was O rough (R)] (Table 3). Of the 109 ONT aEPEC, *espM* was found in 45 isolates (41 %). Among the aEPEC that shared a serogroup with the major EHEC strains, *espM* was found in 23 of 31 (74 %) O26, 4 of 12 (33 %) O103, 2 of 4 (50 %) O111, 25 of 33 (76 %) O145 and 4 of 7 (57 %) O157 isolates; in total 58 of 87 (67 %). Interestingly, *espM* was found in 94 % (15 of 16) of the O55 strains, regardless of serotype. *espT* was found in only 1 (0.8 %) tEPEC strain (O111 : H-) isolated in Spain and in 12 aEPEC strains (2 %) (Table 3).

In order to confirm the absence of *espM* and *espT* from the PCR gene-negative EPEC strains, we screened 50 tEPEC and 100 aEPEC strains with *espM* primers 3 and 4, and *espT* primers 3 and 4, 5 and 6, and 7 and 8. All the isolates remained *espM* and *espT* gene negative in these tests.

### Further characterization of the *espT*-positive strains

**Fig. 1. f1:**
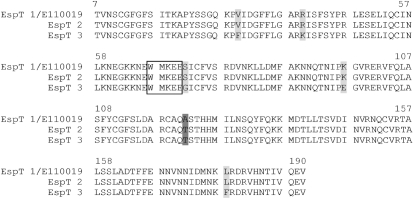
Multiple sequence alignment of representative EspT sequences. The conserved motif WxxxE is boxed. When compared with EspT_E110019_, 8 of the 13 EspT showed 100 % sequence identity (ONT : H-, O49 : H-, O85 : H-, O109 : H9, O111 : H-, O123 : H- (2), O154 : H9) as represented by sequence EspT 1. Two strains belonging to serotype O2 : H49 had 1 amino acid difference, indicated by dark grey (sequence EspT 2), while three strains O104 : H2 (2), ONT : H (7) differed in 6 amino acids, indicated by dark grey and light grey (sequence EspT 3).

Considering that *espT* was found in only 13 of the total 932 isolates tested, we sequenced their amplicons, and characterized their virulence genes implicated in colonization (e.g. intimin type) and pedestal formation (e.g. Tir type and TccP2) as described previously (Garmendia *et al.*, 2005b); strain E110019 was used as a reference strain. Sequencing of *espT* revealed a high level of sequence conservation (Table 4). In the eight strains EspT was identical to that of E110019, defined as group 1 EspT. In two strains we detected a single amino acid difference (group 2) and in three other strains we found 6 amino acids that differed from the EspT of E110019 (group 3) (Fig. 1). All the *espT*-positive strains encoded a Tir that can undergo tyrosine phosphorylation and thus trigger strong actin polymerization *in vitro* via the Nck-N-WASP pathway (Kenny, 1999; Gruenheid *et al.*, 2001). All the strains but two encoded TccP2, which can also activate the N-WASP actin-signalling pathway (Whale *et al.*, 2007). Eight of the *espT*-positive strains (as well as E110019) also encoded EspM.

Intimin typing was performed by sequencing the variable 3′ end of the *eae* gene from the 14 *espT*-positive strains (including E110019) (Table 4) (Blanco *et al.*, 2006b). This revealed known intimin types in 10 strains: *β*1 (3 strains), ε2 (4 strains), *θ*1 (1 strain) and ι1 (2 strains). Importantly, we identified a new intimin variant, *ζ*2, in 4 of the *espT* gene-positive strains (Table 4). We determined the complete nucleotide sequence of two of the new *eae*-*ζ*2 variant genes (accession numbers FM872419 and FM872420 for E110019 and T2381-8, respectively). Using clustal w software for optimal sequence alignment with the known 27 *eae* alleles, we found 98, 92 and 91 % sequence identity with the *eae*-*ζ*1 (AJ271407), *eae*-*α*1 (M58154) and *eae*-*α*2 (AF530555) genes, respectively.

### Conclusions

Our results show that *espM* is found in approximately 96 % of the strains belonging to the major EHEC serogroups: O26, O103, O111, O118, O145 and O157, and in a significantly higher proportion than in other non-O157 EHEC strains (*P*<0.05). Similarly, *espM* was also more commonly found in EPEC serogroups O26, O55, O145 and O157 than in other aEPEC. Among the tEPEC strains *espM* was detected in approximately 69 % of the tested isolates. These results suggest that *espM* is more commonly found in EPEC and EHEC serogroups that are linked to severe human infections. In contrast, *espT* was found only infrequently and only among EPEC strains (1 tEPEC and 12 aEPEC isolates). *espT* was first found in *C. rodentium* and later *in silico* in EPEC E110019, which is of particular interest as it was responsible for a particularly severe diarrhoeal outbreak in Finland in 1987 that affected 650 individuals, including adults, in a school complex and an additional 137 associated household members (Viljanen *et al.*, 1990). Comparing the protein sequences of EspT to that of E110019 showed a high level of conservation, with only three strains encoding EspT that differed in 6 amino acids from the EspT of E110019. At present, it is not clear why *espT* is so rare and what impact EspM and EspT have on EPEC and EHEC infection.

## Figures and Tables

**Table 1. t1:** Distribution of *espM* and *espT* among 151 non-O157 clinical EHEC strains (Spain) The strains were isolated in Spain, except for strain FV10110 O111 : H8, which was isolated in Germany. *espM–espT* negative strains were: ONT : HNT (2), ONT : H5 (1), ONT : H8 (1), ONT : H18 (1), ONT : H39 (1), O2 : H27 (1), O8 : H2 (1), O8 : H19 (1), O15 : H16 (1), O15 : H28 (1), O18 : ND (2), O22 : H42 (1), O55 : H- (1), O63 : HND (2), O64 : H21 (1), O76 : ND (2), O76 : H19 (2), O84 : HNT (1), O84 : HND (1), O91 : H- (2), O98 : H- (1), O104 : HNT (4), O113 : HND (2), O113 : H21 (2), O117 : HND (1), O136 : HND (1), O141 : HND (1), O146 : H- (1), O146 : H21 (4), O148 : HND (2), O148 : H8 (1), O165 : H- (1), O166 : HND (1), O166 : H28 (1), O168 : H8 (1), O174 : H21 (1), O183 : H- (1).

**Serotype (no. of strains)**		**VT type**	***espM***	***espT***
**O**	**H**			
ONT (14)	HND (7), H- (6), H11 (1)	1, 2	4	0
O5	HND (3)	1, 2	2	0
O14	H- (1)	1	1	0
O26 (37)	HND (17), H- (2), H8 (1), H11 (17)	1	36	0
O32	H6 (1)	1	1	0
O69	H21 (1)	1	1	0
O80	HND (1)	1, 2	1	0
O98	H21 (1)	1	1	0
O103 (6)	HND (1), H2 (5)	1, 2	6	0
O111 (10)	HND (5), H- (3), H8 (1)*, H10 (1)	1, 2	9	0
O118 (6)	HND (3), H- (1), H16 (2)	1	6	0
O121 (2)	H19 (1), H40 (1)	2	2	0
O145 (3)	HND (1), H- (2)	1, 2	3	0
O146	HND (10)	1, 2	1	0
O156	H25 (2)	1	2	0
O139, O141	HND (1)	1, 2	1	0

VT, Verocytotoxin. *Strain FV10110 O111 : H8 was isolated in Germany.

**Table 2. t2:** Distribution of *espM* and *espT* among 132 tEPEC strains *espM–espT* negative strains were: ONT : HND (1), ONT : H1, H12 (1), ONT : H8 (1), ONT : H25 (1), O1 : H1, H12 (1), O55 : H8 (1), O86 : H8 (1), O88 : H5 (2), O98 : HND (1), O127 : H40 (3), O131 : H46 (2), O153 : H8 (1), O157 : H45 (1).

**Serotype (no. of strains)**		**Origin**	***espM***	***espT***
**O**	**H**			
ONT (2)	H6 (1), H10 (1)	Bolivia	2	0
O23 (2)	HND (1), H8 (1)	Spain	2	0
O49	H10 (5)	Spain, Bolivia	5	0
O55 (22)	H- (8), H6 (6), H51 (8)	Uruguay, Bolivia, Brazil, UK	19	0
O86 (6)	H- (2), H34 (4)	Bolivia, Brazil	6	0
O88 (17)	H- (1), H6 (1), H25 (15)	Spain, Bolivia	3	0
O103 (3)	H- (1), H7 (2)	Bolivia	3	0
O109	H- (2)	Spain, Chile	1	0
O111 (20)	H- (12), H2 (7), H25 (1)	Spain, Bolivia, Brazil, Uruguay	19	1
O118 (4)	HND (1), H- (1), H5 (2)	Spain, Germany	4	0
O119 (17)	H- (2), H6 (15)	Bolivia, Brazil, Uruguay	16	0
O125	H- (1)	Burundi	1	0
O127	H6 (3)	Bolivia, Brazil	1	0
O132	H8 (1)	Bolivia	1	0
O142 (6)	H6 (2), H34 (4)	Brazil, Spain	4	0
O145	H45 (3)	Brazil	3	0
O153	H11 (1)	Spain	1	0

**Table 3. t3:** Distribution of *espM* and *espT* among 602 aEPEC strains *espM–espT* negative strains were: ONT : H2 (2), ONT : H6 (4), ONT : H9 (1), ONT : H10 (2), ONT : H11, 21, 34 (1), ONT : H19 (2), ONT : H21, 39 (1), ONT : H34 (1), ONT : H40, 43 (2), ONT : H51 (1), O1 : H2 (1), O1 : H11 (1), O1 : H46 (1), O1 : H49 (1), O2ab : H45 (1), O2 : NT (1), O2 : H6 (1), O2 : H16 (1), O2 : H45 (1), O2 : H49 (2), O3 : H4 (1), O5 : H6 (1), O5 : H40 (1), O5 : H49 (1), O6 : H4 (1), O6 : H16 (1), O9 : HND (2), O11 : H16 (2), O12 : HND (1), O13 : H11 (1), O15 : H- (2), O15 : H7 (1), O16 : H- (1), O18 : H7 (1), O18 : H16 (1), O20 : H6 (1), O21 : HND (2), O21 : H15 (1), O23 : HND (1), O24 : H2 (1), O25 : HND (2), O25 : H1 (1), O26 : H2 (1), O28 : HND (1), O33 : HNT (1), O33 : H6 (1), O33 : H34 (1), O34 : H- (1), O41 : H- (1), O49 : H40 (1), O51 : H41 (1), O55 : H40 (1), O56 : H6 (1), O63 : HND (1), O64 : H40 (1), O71 : H1, H12 (2), O76 : H19 (1), O84 : HND (1), O84 : H- (2), O85 : H8 (1), O85 : H49 (1), O86 : HNT (1), O86 : HND (1), O88, O168 : HND (1), O98 : H- (1), O98 : H8 (1), O101 : H33 (2), O103 : H- (3), O103 : H4 (1), O103 : H19 (1), O103 : H40 (1), O104 : H- (2), O105 : HND (1), O105 : H4 (2), O108 : HND (3), O110 : HND (5), O111 : H9 (1), O111 : H38 (1), O112 : H15 (1), O113 : HND (4), O113 : H- (1), O114 : HND (1), O117 : H11 (1), O117 : H40 (2), O121 : HND (1), O123 : HND (1), O123 : H19 (1), O123 : H45 (1), O125 : H6 (3), O127 : HND (1), O127 : H6 (1), O128 : H40 (1), O129 : HND (1), O129 : H- (1), O132 : HND (3), O132 : H5 (3), O137 : HND (3), O137 : H6 (2), O139 : HND (4), O139 : H2 (1), O141 : HND (10), O145 : H2 (1), O146 : H28 (1), O148 : H8 (1), O153 : H11 (1), O153 : H31 (1), O153 : H40 (1), O156 : H4 (1), O156 : H8 (3), O156 : H33 (1), O157 : H16 (2), O159 : HND (1), O164 : H- (1), O167 : H27 (1), O168 : H- (1), O171 : H19 (1), O173 : H- (1), O178 : H6 (1), O180 : HND (1), O180 : H2 (1), R : H28 (1).

**Serotype (no. of strains)**		**Origin**	***espM***	***espT***
**O**	**H**			
ONT (92)	HND (38), HNT (4), H- (24), H4 (3), H7 (4), H8 (3), H11 (4), H23 (1), H33 (2), H40 (6), H45 (3)	Spain, Brazil, Bolivia, Chile		
O1 (3)	H7 (1), H40 (1), H45 (1)	Brazil, Spain	3	0
O2 (11)	ND (3), H40 (8)	Spain	3	2
O4 (4)	ND (2), H- (1), H1 (1)	Spain, Brazil	3	0
O5	ND (7)	Spain	2	0
O6 (2)	ND (1), H19 (1)	Spain	2	0
O8 (5)	HND (3), H11 (1), H19 (1)	Spain	3	0
O10 (5)	HN (3), H- (2)	Spain	4	0
O11	HND (4)	Spain	4	0
O14	H5 (1)	Brazil	1	0
O15 (17)	HND (9), H2 (8)	Spain	12	0
O18	HND (2)	Spain	2	0
O20	HND (2)	Spain	2	0
O22	HND (2)	Spain	2	0
O25	H2 (1)	Spain	1	0
O26 (30)	ND (20), H- (6), H11 (4)	Spain, Brazil	23	0
O28	H28 (1)	Bolivia	1	0
O33	HND (3)	Spain	1	0
O49 (12)	HND (6), H- (3), H2 (1), H10 (2)	Spain, Brazil	6	1
O51 (16)	HND (5), H- (2), H1 (1), H40 (6), H49 (2)	Spain, Brazil	7	0
O52	H10 (1)	Spain	1	0
O57	H7 (3)	Spain	3	0
O55 (16)	HND (4), H- (1), H6 (1), H7 (9), H51 (1)	Spain, Brazil, Bolivia	15	0
O56	H- (1)	Spain	1	0
O63 (6)	H6 (5), H33 (1)	Spain, Brazil	2	0
O64	H25 (2)	Spain	1	0
O70	H2 (1)	Brazil	1	0
O76	HND (3)	Spain	2	0
O80 (9)	ND (5), H2 (4)	Spain	4	0
O82 (2)	HND (1), H10 (1)	Spain	2	0
O85 (13)	HND (5), H- (2), H31 (5), H43 (1)	Spain	4	1
O101 (7)	HND (2), H- (5)	Spain, Brazil	7	0
O103 (12)	HND (11), H2 (1)	Spain	4	0
O104 (4)	HND (1), H2 (2), H12 (1)	Spain, Brazil	2	2
O109	H9 (1)	Brazil	1	1
O111 (2)	HND (1), H- (1)	Spain	2	0
O113	H6 (4)	Spain	1	0
O115 (4)	HND (1), H8 (3)	Spain	3	0
O117	HND (4)	Spain	2	0
O119	HND (2)	Spain	2	0
O120	HND (2)	Spain	1	0
O123	H- (2)	Brazil	2	2
O125	H28 (1)	Spain	1	0
O127	H40 (5)	Spain, Bolivia	1	0
O128 (4)	HND (1), H- (1), H2 (1), H27 (1)	Spain	4	0
O132	H34 (3)	Spain	3	0
O139	H19 (1)	Spain	1	0
O145 (33)		Spain, Germany	25	0
O146	HND (1)	Spain	1	0
O153	HND (15)	Spain	2	0
O154	H9 (1)	Brazil	1	1
O156 (8)	HND (6), H- (2)	Spain	4	0
O157 (5)	ND (2), H7 (3)	Spain	4	0
O162 (4)	H- (2), H21 (1), H33 (1)	Spain, Brazil	3	0
O166	HND (1)	Spain	1	0
O167 (4)	ND (2), H9 (1), H11 (1)	Spain	3	0
O177	H11 (1)	Spain	1	0
O178	H7 (1)	Spain	1	0
O179	H31 (1)	Spain	1	0
O115, O152 (15)	HND (13), H8 (1), H10 (1)	Spain	10	0
R (2)	H11, 21 (1), H28 (1)	Brazil	1	0

**Table 4. t4:** Characterization of the *espT*-positive strains

**Serotype (no. of strains)**	**Origin**	**Pathotype**	***espM***	**Intimin**	**Tir**	**Tccp2**	**GenBank accession no.**
**Group 1**							
O111 : H9 (1)	Finland	aEPEC	*+*	*ζ*2	*α*1	+	
ONT : H- (1)	Brazil	aEPEC	+	ε2	*α*1	+	FM992854
O49 : H- (1)	Brazil	aEPEC	+	*ζ*2	*α*1	+	FM992855
O85 : H- (1)	Brazil	aEPEC	–	ι1	*α*1	+	FM992856
O109 : H9 (1)	Brazil	aEPEC	+	ε2	*α*1	+	FM992857
O111 : H- (1)	Spain	tEPEC	+	*ζ*2	*α*1	+	FM992858
O123 : H- (2)	Brazil	aEPEC	+	ε2	*α*1	+	FM992859
O154 : H9 (1)	Brazil	aEPEC	+	*ζ*2	*α*1	+	FM992860
**Group 2**							
O2 : H49 (1)	Spain	aEPEC	–	*θ*1	*θ*1	+	FM992862
O2 : H49 (1)	Spain	aEPEC	–	ι1	*α*1	–	FM992861
**Group 3**							
ONT : H7 (1)	Brazil	aEPEC	+	*β*1	*β*1	–	FM992863
O104 : H2 (1)	Spain	aEPEC	–	*β*1	*β*1	+	FM992864
O104 : H2 (1)	Brazil	aEPEC	–	*β*1	*β*1	+	FM992865

## Abbreviations

- A/E, attaching and effacing
- aEPEC, atypical enteropathogenic *Escherichia coli*
- EHEC, enterohaemorrhagic *Escherichia coli*
- EPEC, enteropathogenic *Escherichia coli*
- SF, sorbitol fermenting
- tEPEC, typical enteropathogenic *Escherichia coli*
